# Three-centre hydrogen bonds in triphenyl­phosphine oxide–hydro­quinone (1/1)

**DOI:** 10.1107/S1600536807067414

**Published:** 2008-01-04

**Authors:** Rodolfo Moreno-Fuquen, Jaime Valderrama-N, Kenneth Shankland, Francesa P. A. Fabbiani, Anders J. Markvardsen

**Affiliations:** a Departamento de Química, Facultad de Ciencias, Universidad del Valle, Apartado 25360, Santiago de Cali, Colombia; b Departamento de Física, Facultad de Ciencias, Universidad del Valle, Apartado 25360, Santiago de Cali, Colombia; c ISIS Facility, Rutherford Appleton Laboratory, Chilton, Didcot, Oxon OX11 0QX, England

## Abstract

The title cocrystal, C_18_H_15_OP·C_6_H_6_O_2_, belongs to a series of mol­ecular systems based on triphenyl­phosphine *P*-oxide. The O atom of the oxide group acts as an acceptor for hydrogen bonds from OH groups of two hydro­quinone mol­ecules which lie on inversion centres [O⋯O = 2.7451 (17) and 2.681 (2) Å]. The crystal structure is stabilized by weak C—H⋯O hydrogen bonds, forming a *C*_2_^1^(8) chain which runs parallel to the [100] direction.

## Related literature

For related literature, see: Al-Farhan (1992 ▶); Etter (1990 ▶); Fuquen & Lechat (1992 ▶); Wallwork & Powell (1980 ▶).
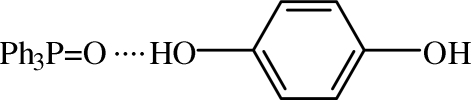


## Experimental

### 

#### Crystal data

C_18_H_15_OP·C_6_H_6_O_2_
*M* *_r_* = 388.38
Triclinic, 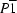

*a* = 8.927 (4) Å
*b* = 9.3576 (10) Å
*c* = 14.459 (4) Å
α = 71.157 (7)°
β = 73.826 (6)°
γ = 62.83 (2)°
*V* = 1004.6 (6) Å^3^
*Z* = 2
Mo *K*α radiation
μ = 0.16 mm^−1^
*T* = 150 (2) K
0.20 × 0.20 × 0.10 mm

#### Data collection

Oxford Diffraction Gemini diffractometer
Absorption correction: multi-scan (*SCALE3 ABSPACK*; Oxford Diffraction, 2006 ▶) *T*_min_ = 0.97, *T*_max_ = 0.98
10150 measured reflections
3560 independent reflections
2837 reflections with *I* > 2σ(*I*)
*R*_int_ = 0.025

#### Refinement

*R*[*F* ^2^ > 2σ(*F* ^2^)] = 0.030
*wR*(*F* ^2^) = 0.077
*S* = 1.11
3560 reflections
261 parameters
H atoms treated by a mixture of independent and constrained refinement
Δρ_max_ = 0.36 e Å^−3^
Δρ_min_ = −0.32 e Å^−3^

### 

Data collection: *CrysAlis CCD* (Oxford Diffraction, 2006 ▶); cell refinement: *CrysAlis RED* (Oxford Diffraction, 2006 ▶); data reduction: *CrysAlis RED*; program(s) used to solve structure: *SHELXS97* (Sheldrick, 1990 ▶); program(s) used to refine structure: *SHELXL97* (Sheldrick, 1997 ▶); molecular graphics: *ORTEP-3 for Windows* (Farrugia, 1997 ▶); software used to prepare material for publication: *PARST95* (Nardelli, 1995 ▶).

## Supplementary Material

Crystal structure: contains datablocks I, global. DOI: 10.1107/S1600536807067414/hg2360sup1.cif

Structure factors: contains datablocks I. DOI: 10.1107/S1600536807067414/hg2360Isup2.hkl

Additional supplementary materials:  crystallographic information; 3D view; checkCIF report

## Figures and Tables

**Table 1:** Hydrogen-bond geometry (Å, °)

*D*—H⋯*A*	*D*—H	H⋯*A*	*D*⋯*A*	*D*—H⋯*A*
O2—H25⋯O1^i^	0.87 (2)	1.87 (2)	2.7451 (17)	175 (2)
O3—H26⋯O1^i^	0.87 (2)	1.82 (2)	2.681 (2)	170 (2)
C3—H3⋯O3^ii^	0.95	2.54	3.300 (2)	137

Symmetry codes: (i) 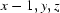; (ii) 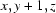.

**Fig. 1.**
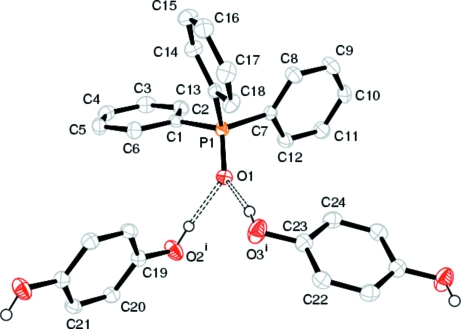
An ORTEP-3 (Farrugia, 1997) plot of the title compound with the atomic labelling scheme. Displacement ellipsoids are drawn at the 50% probability level. Ring H-atoms were omitted for clarity. The dashed line indicates a hydrogen bond. [Symmetry code: (i) 1 + x, y, z]

**Fig. 2.**
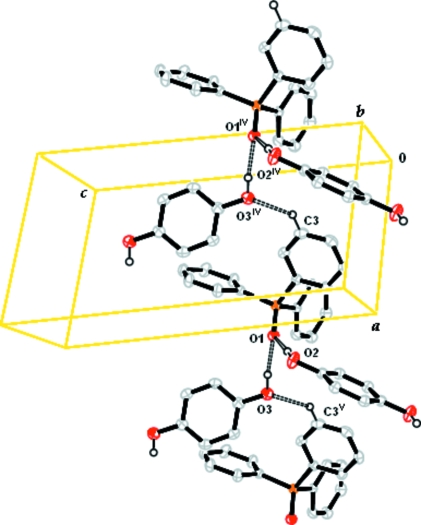
View of the crystal structure of (I), showing the O—H···O and C—H···O interactions along [100] direction. [Symmetry codes: (iv) x, 1 + y, z (v) 1 + x, 1 + y, z]

## supplementary crystallographic information

### Comment

The title compound, C_18_H_15_OP.C_6_H_6_O_2_, belongs to a series of molecular systems based on triphenylphosphine P-oxide (TPPO) with diverse hydrogen-bond donors (Fuquen *et al.*, 1992). In order to expand the crystallographic information of the TPPO complexes, to study the hydrogen bond character of the complex, and to analize its supramolecular arrangement, the structure determination of TPPO + hydroquinone (HQ), (I), system was undertaken. The free HQ molecule in the more stable form at room temperature (Wallwork & Powell, 1980) and the free TPPO molecule (Al-Farhan, 1992) can be taken as a reference systems to compare with the structural characteristics of (I). A displacement ellipsoid plot of the title hydrogen-bonded complex (I), showing the atomic numbering scheme is given in Fig. 1. The O1 atom of the P-oxide group of TPPO acts as an acceptor for hydrogen bonds from O—H groups of two hydroquinone molecules [O1···O2, 2.7451 (17), O1···O3, 2.681 (2) Å and O1···H25—O2, O1···H26—O3 angles of 175 (2) and 170 (2)° respectively, (Table 2)]. These two HQ molecules are each disposed about a centre of symmetry. The title molecule shows a H25—O1—H26 bond angle close to the right angle, seeking an orientation with the minor repulsion between the rings of the molecule. The presence of the three centre hydrogen bond at O1 induces the lengthening of P—O bond length from 1.479 (2) Å in free TPPO molecule (Al-Farhan, 1992) to 1.5016 (13) Å in (I). Other bond lengths and angles of TPPO and HQ remain similar in the complex. The title molecules of (I) are additionally linked by C—H···O hydrogen bonds. Indeed, atom C3 in the molecule at (*x*, *y*, *z*) acts as a hydrogen-bond donor to O3^iv^ atom in the molecule at (*x*, 1 + *y*, *z*), so generating a C_2_^1^(8) chain (Etter, 1990) which is running parallel to [100] direction (Fig. 2, Supp.material). Other significant intermolecular hydrogen bonds are not observed in the crystalline structure.

### Experimental

Crystals of the title compound (I), were obtained by slow evaporation of equimolecular quantities of HQ (1.826 g, 0.017 mol) and TPPO (4.725 g) in 150 ml of dry acetonitrile. After three days, colourless plates of a good quality suitable for X-ray analysis were obtained. Its melting point is 425 (1) K.

### Refinement

All non-hydrogen atoms were identified by direct methods and the positions of all the hydrogen atoms were obtained from the use of difference Fourier maps. In the final refinement, all hydrogen atoms were constrained to geometrically sensible positions with a riding model (*SHELX97*), C—H= 0.95 Å, and *U*_iso_(H)= 1.5*U*_eq_(C), apart from H25 and H26, which were allowed to refine freely.

### Crystal data

**Table:**

C_18_H_15_OP·C_6_H_6_O_2_	*Z* = 2
*M**_r_* = 388.38	*F*_000_ = 408
Triclinic, *P*1	*D*_x_ = 1.284 Mg m^−^^3^
Hall symbol: -P 1	Melting point: 425(1) K
*a* = 8.927 (4) Å	Mo *K*α radiation λ = 0.71073 Å
*b* = 9.3576 (10) Å	Cell parameters from 6084 reflections
*c* = 14.459 (4) Å	θ = 2.5–28.6º
α = 71.157 (7)º	µ = 0.16 mm^−^^1^
β = 73.826 (6)º	*T* = 150 (2) K
γ = 62.83 (2)º	Plate, colourless
*V* = 1004.6 (6) Å^3^	0.20 × 0.20 × 0.10 mm

### Data collection

**Table:**

Oxford Diffraction Gemini diffractometer	3560 independent reflections
Radiation source: fine-focus sealed tube	2837 reflections with *I* > 2σ(*I*)
Monochromator: graphite	*R*_int_ = 0.025
Detector resolution: 15.975 pixels mm^-1^	θ_max_ = 25.0º
*T* = 150(2) K	θ_min_ = 2.5º
ω and π scans	*h* = −10→10
Absorption correction: multi-scan[empirical (using intensity measurements) absorption correction using spherical harmonics, implemented in SCALE3 ABSPACK scaling algorithm (Oxford Diffraction, 2006)]	*k* = −11→11
*T*_min_ = 0.97, *T*_max_ = 0.98	*l* = −14→17
10150 measured reflections	

### Refinement

**Table:**

Refinement on *F*^2^	Secondary atom site location: difference Fourier map
Least-squares matrix: full	Hydrogen site location: inferred from neighbouring sites
*R*[*F*^2^ > 2σ(*F*^2^)] = 0.030	H atoms treated by a mixture of independent and constrained refinement
*wR*(*F*^2^) = 0.077	*w* = 1/[σ^2^(*F*_o_^2^) + (0.0364*P*)^2^ + 0.182*P*] where *P* = (*F*_o_^2^ + 2*F*_c_^2^)/3
*S* = 1.11	(Δ/σ)_max_ < 0.001
3560 reflections	Δρ_max_ = 0.36 e Å^−^^3^
261 parameters	Δρ_min_ = −0.32 e Å^−^^3^
Primary atom site location: structure-invariant direct methods	Extinction correction: none

### Special details

**Table:**

Geometry. All e.s.d.'s (except the e.s.d. in the dihedral angle between two l.s. planes) are estimated using the full covariance matrix. The cell e.s.d.'s are taken into account individually in the estimation of e.s.d.'s in distances, angles and torsion angles; correlations between e.s.d.'s in cell parameters are only used when they are defined by crystal symmetry. An approximate (isotropic) treatment of cell e.s.d.'s is used for estimating e.s.d.'s involving l.s. planes.
Refinement. Refinement of *F*^2^ against ALL reflections. The weighted *R*-factor *wR* and goodness of fit *S* are based on *F*^2^, conventional *R*-factors *R* are based on *F*, with *F* set to zero for negative *F*^2^. The threshold expression of *F*^2^ > σ(*F*^2^) is used only for calculating *R*-factors(gt) *etc*. and is not relevant to the choice of reflections for refinement. *R*-factors based on *F*^2^ are statistically about twice as large as those based on *F*, and *R*- factors based on ALL data will be even larger.

### Fractional atomic coordinates and isotropic or equivalent isotropic displacement parameters (Å^2^)

**Table:**

	*x*	*y*	*z*	*U*_iso_*/*U*_eq_	
P1	0.95138 (5)	0.39823 (5)	0.27946 (3)	0.01905 (11)	
O1	1.12889 (12)	0.37744 (12)	0.27829 (7)	0.0236 (2)	
O2	0.31558 (16)	0.56334 (14)	0.18170 (8)	0.0330 (3)	
O3	0.43737 (16)	0.13741 (16)	0.30698 (8)	0.0390 (3)	
C19	0.40534 (19)	0.52905 (18)	0.09190 (10)	0.0225 (3)	
C13	0.95218 (18)	0.23915 (17)	0.23485 (10)	0.0198 (3)	
C18	1.0897 (2)	0.08595 (18)	0.24493 (11)	0.0265 (4)	
H18	1.1811	0.0670	0.2750	0.040*	
C24	0.3343 (2)	0.09996 (18)	0.48323 (11)	0.0269 (4)	
H24	0.2203	0.1685	0.4719	0.040*	
C21	0.59840 (19)	0.58783 (18)	−0.05380 (11)	0.0240 (3)	
H21	0.6654	0.6487	−0.0905	0.036*	
C10	0.6658 (2)	0.37566 (19)	0.59682 (11)	0.0277 (4)	
H10	0.6091	0.3701	0.6632	0.042*	
C14	0.81782 (19)	0.26512 (19)	0.19154 (11)	0.0254 (3)	
H14	0.7230	0.3688	0.1848	0.038*	
C1	0.84087 (18)	0.59192 (17)	0.19937 (10)	0.0195 (3)	
C8	0.7437 (2)	0.29020 (19)	0.44432 (11)	0.0274 (4)	
H8	0.7401	0.2258	0.4065	0.041*	
C7	0.83224 (18)	0.39133 (17)	0.40226 (10)	0.0200 (3)	
C6	0.9123 (2)	0.61763 (18)	0.09972 (11)	0.0250 (3)	
H6	1.0129	0.5328	0.0765	0.037*	
C3	0.6205 (2)	0.86657 (19)	0.16682 (12)	0.0303 (4)	
H3	0.5207	0.9524	0.1897	0.046*	
C20	0.50465 (19)	0.61583 (18)	0.03765 (11)	0.0237 (3)	
H20	0.5086	0.6953	0.0633	0.035*	
C9	0.6605 (2)	0.2829 (2)	0.54123 (11)	0.0310 (4)	
H9	0.5998	0.2140	0.5695	0.047*	
C16	0.9609 (2)	−0.01175 (19)	0.16781 (12)	0.0306 (4)	
H16	0.9642	−0.0972	0.1443	0.046*	
C5	0.8378 (2)	0.76576 (19)	0.03425 (11)	0.0281 (4)	
H5	0.8866	0.7821	−0.0336	0.042*	
C12	0.8361 (2)	0.48507 (19)	0.45913 (11)	0.0273 (4)	
H12	0.8958	0.5550	0.4312	0.041*	
C15	0.8228 (2)	0.1395 (2)	0.15826 (12)	0.0308 (4)	
H15	0.7311	0.1572	0.1288	0.046*	
C17	1.0939 (2)	−0.03884 (19)	0.21136 (12)	0.0310 (4)	
H17	1.1881	−0.1430	0.2183	0.046*	
C22	0.6308 (2)	−0.02875 (18)	0.42058 (11)	0.0262 (4)	
H22	0.7207	−0.0489	0.3663	0.039*	
C2	0.69447 (19)	0.71779 (18)	0.23265 (11)	0.0247 (3)	
H2	0.6450	0.7021	0.3004	0.037*	
C23	0.4649 (2)	0.07154 (18)	0.40370 (11)	0.0258 (4)	
C11	0.7535 (2)	0.4765 (2)	0.55590 (11)	0.0306 (4)	
H11	0.7570	0.5401	0.5943	0.046*	
C4	0.6920 (2)	0.88961 (19)	0.06822 (12)	0.0284 (4)	
H4	0.6407	0.9912	0.0234	0.043*	
H25	0.251 (3)	0.509 (3)	0.2108 (15)	0.059 (6)*	
H26	0.332 (3)	0.209 (3)	0.3043 (14)	0.055 (6)*	

### Atomic displacement parameters (Å^2^)

**Table:**

	*U*^11^	*U*^22^	*U*^33^	*U*^12^	*U*^13^	*U*^23^
P1	0.0199 (2)	0.0200 (2)	0.0180 (2)	−0.00858 (16)	−0.00232 (15)	−0.00511 (15)
O1	0.0217 (6)	0.0269 (6)	0.0236 (6)	−0.0118 (5)	−0.0035 (4)	−0.0044 (4)
O2	0.0419 (7)	0.0360 (7)	0.0268 (6)	−0.0243 (6)	0.0089 (5)	−0.0137 (5)
O3	0.0303 (7)	0.0430 (7)	0.0287 (7)	−0.0009 (6)	−0.0079 (5)	−0.0079 (5)
C19	0.0222 (8)	0.0216 (7)	0.0213 (8)	−0.0071 (7)	−0.0030 (6)	−0.0050 (6)
C13	0.0221 (8)	0.0213 (7)	0.0157 (7)	−0.0094 (6)	−0.0019 (6)	−0.0036 (6)
C18	0.0281 (9)	0.0236 (8)	0.0263 (8)	−0.0063 (7)	−0.0097 (7)	−0.0056 (6)
C24	0.0214 (8)	0.0224 (8)	0.0357 (9)	−0.0062 (7)	−0.0060 (7)	−0.0077 (7)
C21	0.0226 (8)	0.0233 (8)	0.0275 (9)	−0.0122 (7)	−0.0038 (7)	−0.0035 (6)
C10	0.0300 (9)	0.0298 (9)	0.0183 (8)	−0.0113 (7)	0.0001 (7)	−0.0036 (7)
C14	0.0242 (9)	0.0232 (8)	0.0288 (9)	−0.0076 (7)	−0.0063 (7)	−0.0071 (6)
C1	0.0211 (8)	0.0205 (7)	0.0210 (8)	−0.0111 (6)	−0.0025 (6)	−0.0064 (6)
C8	0.0330 (9)	0.0284 (8)	0.0258 (9)	−0.0170 (7)	0.0010 (7)	−0.0103 (7)
C7	0.0203 (8)	0.0191 (7)	0.0199 (8)	−0.0075 (6)	−0.0044 (6)	−0.0032 (6)
C6	0.0261 (9)	0.0241 (8)	0.0237 (8)	−0.0086 (7)	−0.0023 (7)	−0.0077 (6)
C3	0.0247 (9)	0.0232 (8)	0.0414 (10)	−0.0052 (7)	−0.0054 (7)	−0.0119 (7)
C20	0.0259 (8)	0.0205 (7)	0.0268 (8)	−0.0099 (7)	−0.0053 (7)	−0.0062 (6)
C9	0.0366 (10)	0.0299 (9)	0.0288 (9)	−0.0211 (8)	0.0051 (7)	−0.0066 (7)
C16	0.0424 (10)	0.0259 (9)	0.0303 (9)	−0.0175 (8)	−0.0048 (8)	−0.0099 (7)
C5	0.0356 (10)	0.0302 (9)	0.0209 (8)	−0.0169 (8)	−0.0047 (7)	−0.0032 (7)
C12	0.0335 (9)	0.0325 (9)	0.0238 (8)	−0.0206 (8)	−0.0017 (7)	−0.0075 (7)
C15	0.0324 (9)	0.0339 (9)	0.0339 (9)	−0.0162 (8)	−0.0090 (7)	−0.0099 (7)
C17	0.0349 (10)	0.0202 (8)	0.0325 (9)	−0.0047 (7)	−0.0080 (8)	−0.0066 (7)
C22	0.0235 (8)	0.0236 (8)	0.0297 (9)	−0.0089 (7)	0.0003 (7)	−0.0084 (7)
C2	0.0220 (8)	0.0256 (8)	0.0260 (8)	−0.0095 (7)	−0.0001 (7)	−0.0086 (7)
C23	0.0281 (9)	0.0205 (8)	0.0288 (9)	−0.0088 (7)	−0.0064 (7)	−0.0055 (6)
C11	0.0384 (10)	0.0366 (9)	0.0242 (9)	−0.0194 (8)	−0.0024 (7)	−0.0118 (7)
C4	0.0323 (9)	0.0207 (8)	0.0350 (10)	−0.0111 (7)	−0.0154 (8)	−0.0007 (7)

### Geometric parameters (Å, °)

**Table:**

P1—O1	1.5016 (13)	C8—C9	1.387 (2)
P1—C7	1.7957 (15)	C8—C7	1.391 (2)
P1—C13	1.8000 (14)	C8—H8	0.9500
P1—C1	1.8020 (15)	C7—C12	1.399 (2)
O2—C19	1.3732 (18)	C6—C5	1.386 (2)
O2—H25	0.87 (2)	C6—H6	0.9500
O3—C23	1.3756 (19)	C3—C4	1.381 (2)
O3—H26	0.87 (2)	C3—C2	1.391 (2)
C19—C20	1.385 (2)	C3—H3	0.9500
C19—C21^i^	1.391 (2)	C20—H20	0.9500
C13—C18	1.393 (2)	C9—H9	0.9500
C13—C14	1.394 (2)	C16—C17	1.382 (2)
C18—C17	1.384 (2)	C16—C15	1.384 (2)
C18—H18	0.9500	C16—H16	0.9500
C24—C23	1.386 (2)	C5—C4	1.383 (2)
C24—C22^ii^	1.389 (2)	C5—H5	0.9500
C24—H24	0.9500	C12—C11	1.384 (2)
C21—C20	1.387 (2)	C12—H12	0.9500
C21—C19^i^	1.391 (2)	C15—H15	0.9500
C21—H21	0.9500	C17—H17	0.9500
C10—C11	1.382 (2)	C22—C23	1.384 (2)
C10—C9	1.382 (2)	C22—C24^ii^	1.389 (2)
C10—H10	0.9500	C22—H22	0.9500
C14—C15	1.386 (2)	C2—H2	0.9500
C14—H14	0.9500	C11—H11	0.9500
C1—C2	1.392 (2)	C4—H4	0.9500
C1—C6	1.395 (2)		
			
O1—P1—C7	111.26 (6)	C1—C6—H6	119.7
O1—P1—C13	111.76 (7)	C4—C3—C2	120.06 (15)
C7—P1—C13	107.64 (7)	C4—C3—H3	120.0
O1—P1—C1	110.35 (7)	C2—C3—H3	120.0
C7—P1—C1	109.06 (7)	C19—C20—C21	120.68 (14)
C13—P1—C1	106.61 (7)	C19—C20—H20	119.7
C19—O2—H25	113.3 (14)	C21—C20—H20	119.7
C23—O3—H26	110.4 (13)	C10—C9—C8	120.17 (15)
O2—C19—C20	117.57 (13)	C10—C9—H9	119.9
O2—C19—C21^i^	123.33 (14)	C8—C9—H9	119.9
C20—C19—C21^i^	119.10 (14)	C17—C16—C15	120.20 (14)
C18—C13—C14	119.35 (14)	C17—C16—H16	119.9
C18—C13—P1	119.06 (11)	C15—C16—H16	119.9
C14—C13—P1	121.58 (11)	C4—C5—C6	119.69 (15)
C17—C18—C13	120.39 (15)	C4—C5—H5	120.2
C17—C18—H18	119.8	C6—C5—H5	120.2
C13—C18—H18	119.8	C11—C12—C7	120.35 (14)
C23—C24—C22^ii^	120.31 (15)	C11—C12—H12	119.8
C23—C24—H24	119.8	C7—C12—H12	119.8
C22^ii^—C24—H24	119.8	C16—C15—C14	120.20 (15)
C20—C21—C19^i^	120.21 (14)	C16—C15—H15	119.9
C20—C21—H21	119.9	C14—C15—H15	119.9
C19^i^—C21—H21	119.9	C16—C17—C18	119.93 (15)
C11—C10—C9	120.09 (14)	C16—C17—H17	120.0
C11—C10—H10	120.0	C18—C17—H17	120.0
C9—C10—H10	120.0	C23—C22—C24^ii^	120.06 (14)
C15—C14—C13	119.93 (14)	C23—C22—H22	120.0
C15—C14—H14	120.0	C24^ii^—C22—H22	120.0
C13—C14—H14	120.0	C3—C2—C1	120.11 (14)
C2—C1—C6	119.13 (14)	C3—C2—H2	119.9
C2—C1—P1	123.52 (11)	C1—C2—H2	119.9
C6—C1—P1	117.28 (11)	O3—C23—C22	117.62 (14)
C9—C8—C7	120.29 (14)	O3—C23—C24	122.74 (14)
C9—C8—H8	119.9	C22—C23—C24	119.63 (14)
C7—C8—H8	119.9	C10—C11—C12	120.10 (14)
C8—C7—C12	119.00 (14)	C10—C11—H11	120.0
C8—C7—P1	122.40 (11)	C12—C11—H11	120.0
C12—C7—P1	118.52 (11)	C3—C4—C5	120.41 (14)
C5—C6—C1	120.60 (14)	C3—C4—H4	119.8
C5—C6—H6	119.7	C5—C4—H4	119.8
			
O1—P1—C13—C18	27.30 (14)	P1—C1—C6—C5	−177.79 (12)
C7—P1—C13—C18	−95.15 (13)	O2—C19—C20—C21	−179.84 (13)
C1—P1—C13—C18	147.95 (12)	C21^i^—C19—C20—C21	0.5 (2)
O1—P1—C13—C14	−153.41 (12)	C19^i^—C21—C20—C19	−0.5 (2)
C7—P1—C13—C14	84.14 (13)	C11—C10—C9—C8	0.2 (2)
C1—P1—C13—C14	−32.76 (14)	C7—C8—C9—C10	−0.3 (2)
C14—C13—C18—C17	0.7 (2)	C1—C6—C5—C4	0.5 (2)
P1—C13—C18—C17	179.98 (12)	C8—C7—C12—C11	0.3 (2)
C18—C13—C14—C15	−0.6 (2)	P1—C7—C12—C11	−176.59 (12)
P1—C13—C14—C15	−179.84 (12)	C17—C16—C15—C14	0.6 (2)
O1—P1—C1—C2	−117.60 (13)	C13—C14—C15—C16	−0.1 (2)
C7—P1—C1—C2	4.90 (15)	C15—C16—C17—C18	−0.5 (2)
C13—P1—C1—C2	120.85 (13)	C13—C18—C17—C16	−0.2 (2)
O1—P1—C1—C6	59.36 (13)	C4—C3—C2—C1	0.1 (2)
C7—P1—C1—C6	−178.14 (11)	C6—C1—C2—C3	0.4 (2)
C13—P1—C1—C6	−62.18 (13)	P1—C1—C2—C3	177.30 (11)
C9—C8—C7—C12	0.0 (2)	C24^ii^—C22—C23—O3	−179.31 (14)
C9—C8—C7—P1	176.74 (12)	C24^ii^—C22—C23—C24	−0.2 (2)
O1—P1—C7—C8	−129.66 (13)	C22^ii^—C24—C23—O3	179.27 (14)
C13—P1—C7—C8	−6.90 (15)	C22^ii^—C24—C23—C22	0.2 (2)
C1—P1—C7—C8	108.39 (13)	C9—C10—C11—C12	0.0 (2)
O1—P1—C7—C12	47.07 (13)	C7—C12—C11—C10	−0.3 (2)
C13—P1—C7—C12	169.82 (12)	C2—C3—C4—C5	−0.4 (2)
C1—P1—C7—C12	−74.88 (13)	C6—C5—C4—C3	0.1 (2)
C2—C1—C6—C5	−0.7 (2)		

Symmetry codes: (i) −*x*+1, −*y*+1, −*z*; (ii) −*x*+1, −*y*, −*z*+1.

### Hydrogen-bond geometry (Å, °)

**Table:**

*D*—H···*A*	*D*—H	H···*A*	*D*···*A*	*D*—H···*A*
O2—H25···O1^iii^	0.87 (2)	1.87 (2)	2.7451 (17)	175 (2)
O3—H26···O1^iii^	0.87 (2)	1.82 (2)	2.681 (2)	170 (2)
C3—H3···O3^iv^	0.95	2.54	3.300 (2)	137

Symmetry codes: (iii) *x*−1, *y*, *z*; (iv) *x*, *y*+1, *z*.
